# Fighting for recovery on multiple fronts: The past, present, and future of clinical trials for spinal cord injury

**DOI:** 10.3389/fncel.2022.977679

**Published:** 2022-09-07

**Authors:** Valerie A. Dietz, Nolan Roberts, Katelyn Knox, Sherilynne Moore, Michael Pitonak, Chris Barr, Jesus Centeno, Scott Leininger, Kent C. New, Peter Nowell, Matthew Rodreick, Cedric G. Geoffroy, Argyrios Stampas, Jennifer N. Dulin

**Affiliations:** ^1^Department of Biology, Texas A&M University, College Station, TX, United States; ^2^Unite 2 Fight Paralysis, Minneapolis, MN, United States; ^3^Department of Neuroscience and Experimental Therapeutics, Texas A&M University, College Station, TX, United States; ^4^Texas A&M Institute for Neuroscience, Texas A&M University, College Station, TX, United States; ^5^Department of Physical Medicine and Rehabilitation, UTHealth Houston McGovern Medical School, Houston, TX, United States

**Keywords:** clinical trial, spinal cord injury, systematic analysis, trends, outcomes, interventions

## Abstract

Through many decades of preclinical research, great progress has been achieved in understanding the complex nature of spinal cord injury (SCI). Preclinical research efforts have guided and shaped clinical trials, which are growing in number by the year. Currently, 1,149 clinical trials focused on improving outcomes after SCI are registered in the U.S. National Library of Medicine at ClinicalTrials.gov. We conducted a systematic analysis of these SCI clinical trials, using publicly accessible data downloaded from ClinicalTrials.gov. After extracting all available data for these trials, we categorized each trial according to the types of interventions being tested and the types of outcomes assessed. We then evaluated clinical trial characteristics, both globally and by year, in order to understand the areas of growth and change over time. With regard to clinical trial attributes, we found that most trials have low enrollment, only test single interventions, and have limited numbers of primary outcomes. Some gaps in reporting are apparent; for instance, over 75% of clinical trials with “Completed” status do not have results posted, and the Phase of some trials is incorrectly classified as “Not applicable” despite testing a drug or biological compound. When analyzing trials based on types of interventions assessed, we identified the largest representation in trials testing rehab/training/exercise, neuromodulation, and behavioral modifications. Most highly represented primary outcomes include motor function of the upper and lower extremities, safety, and pain. The most highly represented secondary outcomes include quality of life and pain. Over the past 15 years, we identified increased representation of neuromodulation and rehabilitation trials, and decreased representation of drug trials. Overall, the number of new clinical trials initiated each year continues to grow, signifying a hopeful future for the clinical treatment of SCI. Together, our work provides a comprehensive glimpse into the past, present, and future of SCI clinical trials, and suggests areas for improvement in clinical trial reporting.

## Introduction

Spinal cord injury (SCI) is a devastating event, typically resulting in lifelong neurological deficits, which affects an estimated 253,000–378,000 persons in the US alone (National Spinal Cord Injury Statistical Center, 2022). Individuals living with SCI and their loved ones face physical, emotional, social, and financial strain. It is estimated that the lifetime cost of SCI ranges from $1.2 to $5.4 million USD per person, with 30% of people undergoing re-hospitalizations one or more times during any given year following injury (National Spinal Cord Injury Statistical Center, 2022). To date, a large number of clinical trials have been initiated in an effort to improve the lives of individuals with SCI. However, there remain no FDA-approved treatments that can even partially improve neurological dysfunction after injury (Ahuja et al., 2016, 2017a, 2020; Elizei and Kwon, 2017; Hachem et al., 2017). In recent years, the establishment of various animal models has redefined our understanding of the mechanisms underlying SCI pathophysiology (Jakeman et al., 2000; Metz et al., 2000; Basso, 2004; Iwanami et al., 2005; Nout et al., 2012; Cheriyan et al., 2014; Kwon et al., 2015; Sharif-Alhoseini et al., 2017; Alizadeh et al., 2019; Fouad et al., 2020b). In addition, novel engineering applications ranging from cellular reprogramming (Fehlings and Vawda, 2011; Khazaei et al., 2016; Bartlett et al., 2020), to the development of sophisticated technology (Collinger et al., 2013; Courtine and Sofroniew, 2019; Squair et al., 2021), have opened new promising therapeutic avenues.

Since 2016, the National Institutes of Health has spent over $530 million on SCI research, and a substantial portion of that has gone toward supporting SCI clinical studies. Indeed, in 2021 more than 25% of NIH-funded projects related to spinal cord injury involved human subjects as reported by report.nih.gov/funding/categorical-spending#/. While there is still no FDA-approved, proven effective treatment for SCI, some clinical studies have shown great promise, and research priorities of individuals living with SCI have been identified (Anderson, 2004). There have been several excellent reviews published discussing advances in key areas of SCI therapeutics, such as stem cell transplantation and neuromodulation (Hawryluk et al., 2008; Gensel et al., 2011; James et al., 2018; Hofer and Schwab, 2019; Bartlett et al., 2020; Platt et al., 2020). However, these reviews typically focus on outcomes and not general conclusions about the priorities, or evolution, of SCI clinical trials. To address this, we have conducted a systematic review of 1,149 SCI clinical trials using data extracted from ClinicalTrials.gov and annotated by a team of investigators. We reviewed clinical trial characteristics including enrollment, phase, results, status, types and numbers of interventions and primary/secondary outcomes, as well as trends over time for the past 15 years. Collectively, this data provides the first comprehensive, systematic analysis of spinal cord injury clinical trials that will be of broad use for researchers, community members, and clinicians. Ultimately, the insights gained from this information highlight the need to continue pushing toward therapeutic interventions in such a way that is more efficient, held to higher reporting standards, and is overall more informative to the broad community.

## Methods

### Search parameters and exclusion criteria

On January 10, 2022, a search was performed on ClinicalTrials.gov using “spinal cord injuries” as the keyword under the “Condition or disease” category. This broad search resulted in 1,411 clinical trials. We downloaded and exported all 1,411 studies with all available data columns as tab-delimited text files. The exported ‘raw' data included the following data categories: Rank, NCT Number, Title, Acronym, Status, Study Results, Conditions, Interventions, Outcome Measures, Sponsor/Collaborators, Gender, Age, Phases, Enrollment, Funded Bys, Study Type, Study Designs, Other IDs, Start Date, Primary Completion Date, Completion Date, First Posted, Results First Posted, Last Update Posted, Locations, Study Documents, and URL. Data was reviewed, classified, and annotated by a team of six investigators (V.A.D., N.R., K.K., S.M., M.P., J.N.D.), with each clinical trial listing reviewed by at least two independent investigators. Any discrepancies during this process were resolved through consultation between the reviewing investigators and a third reviewer from the team.

**Figure 1:**
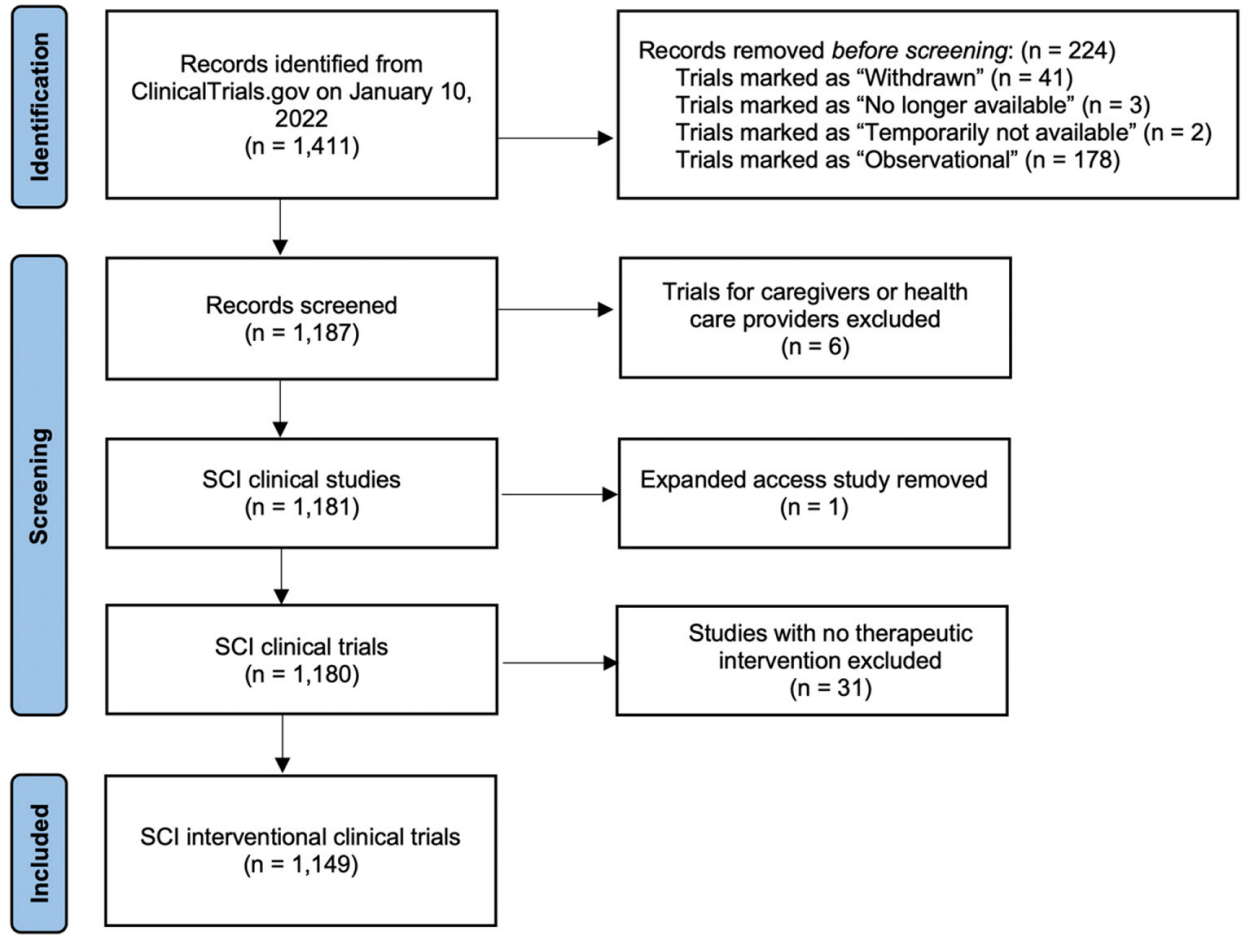
PRISMA flow diagram of the search strategy used in this study. SCI, spinal cord injury (Page et al., 2021).

Prior to screening, we first excluded listings with Status that was classified as “Withdrawn,” “No longer available,” or “Temporarily unavailable,” as well as trials that were classified as Study Type “Observational” (Figure 1). Clinical trials with the status “Withdrawn” are defined by ClinicalTrials.gov as a trial that ended early before enrolling its first patient. Next, we excluded clinical trial listings that were targeted toward caregivers or healthcare providers, but not individuals with SCI. We removed one listing that was not a clinical trial but rather an expanded access program for an investigational new drug. Finally, we refined the list of clinical trials to exclude those that did not include a therapeutic intervention (intended to have a therapeutic or beneficial effect on patients with SCI), as judged by the investigating team. This led to the exclusion of trials that were focused on generation or validation of a diagnostic tool, identification of biomarkers, or development of an intervention without testing the effects of the intervention. A total of 262 clinical trial listings were excluded based on these criteria, leaving 1,149 clinical trials used for analysis.

### Clinical trial annotation and classification

We generated categories for interventions and outcomes based on common themes that emerged upon reviewing the list of clinical trials. Categories are defined with examples in Tables 1, 2. For intervention type, we formulated 14 unique categories: Acupuncture/needle therapy, Antibody therapy, Assistive/wearable technology, Behavioral, Biomaterials transplantation, Cell or tissue transplantation, Drug, Implanted/internal medical device, Nerve transfer/tendon transfer, Neuromodulation/electrical stimulation, Radiation therapy/laser therapy, Rehab/training/exercise, Surgical intervention/medical procedure, and Other (Table 1). The “Drug” category was further broken down into 15 subcategories according to the class or group of drug being tested. For types of primary and secondary outcome measures, we formulated 37 unique categories: Activity Level, Autonomic dysreflexia, Biomechanics/kinematics, Bladder function/bladder health, Blood pressure/cardiovascular function, Body mass/composition, Bone health, Bowel function/bowel health, Cognition, Depression/Anxiety, Employment/occupational performance, Fatigue, Fertility/sexual function, Independence, Medical imaging, Metabolism, Motor (lower extremities/locomotor function), Motor (not specified), Motor (trunk), Motor (upper extremities/hand function), Muscle and/or nerve function, Neurological score, Pain, Pharmacokinetics, Pressure injuries/pressure sores/wound healing, Psychological/social, Pulmonary function/breathing/cough, Quality of life, Safety, Sensory function, Sleep, Spasticity, Survival, Thermoregulation, Usability/feasibility/satisfaction of the intervention, Wheelchair propulsion/mobility, and Other (Table 2).

**Table 1:** Intervention categories.

**Intervention type**	**Definition and examples**
Acupuncture/needle therapy	**Definition**: Puncturing or pricking the skin with needles as a therapeutic practice.
Antibody therapy	**Definition**: Treatment with a monoclonal antibody.
Assistive/wearable technology	**Definition**: Any technology that is worn on the person or used by the person, which does not provide electrical stimulation or directly modulate the nervous system. **Examples**: Wearable garments, robotic gloves, prosthetics, orthoses, vibration/mechanical stimulation devices, CPAP, tongue-control devices, exoskeleton, adaptive robotic devices, adapted furniture, adapted environment.
Behavioral	**Definition**: Interventions that require the individual to modify their behavior, either short-term (during a study visit) or long-term (at home throughout the duration of the study), to produce a desired therapeutic effect. **Examples**: Phone apps, wellness or therapy groups, telemedicine programs, counseling programs, music therapy, educational programs, community programs, modifying diet or exercise routines, self-management routines, cognitive behavioral therapy, hypnosis, virtual reality programs presenting a different environment, visual illusions (e.g., phantom hand).
Biomaterials transplantation	**Definition**: Transplantation of a bioengineered material or biological scaffold, which may or may not contain cells or tissue, into the spinal cord. **Examples**: NeuroRegen scaffold, polyethylene glycol, hyaluronic acid.
Cell or tissue transplantation	**Definition**: Transplantation of living tissue or cells, either into the spinal cord or somewhere else into the body. This excludes biomaterials. **Examples**: Neural stem cells, bone marrow stem cells, mesenchymal stem cells, umbilical cord blood-derived cells, Schwann cells, oligodendrocyte precursor cells.
Drug	**Definition**: A pharmaceutical compound, medicine, supplement, or biological compound that is ingested or delivered into the body. Definitions for some of the subcategories are included below. **Subcategories**: **Adenosine receptor agonist/antagonist**: A compound that modulates activity of adenosine receptors. **Adrenergic receptor agonist/antagonist**: A compound that modulates activity of adrenergic receptors. **Anti-inflammatory**: Non-steroidal anti-inflammatory drugs. **Antibiotic** **Botulinum toxin** **Cannabinoid**: Natural or synthetic compounds within the cannabinoid family. **Growth factor**: Recombinant growth factor such as FGF, EGF, NGF, BDNF. **Herbal/natural/supplement**: Includes vitamins, homeopathic treatments, probiotics, dietary supplements, herbal supplements. **Hormone** **Lidocaine** **Neuromodulatory**: A drug, not falling into the other subcategories, that exerts a direct effect on the nervous system; examples include neurotransmitter reuptake inhibitor or a compound that mimics the effect of a neurotransmitter. **Opioid** **Statin** **Vasoactive**: A drug that exerts effects on blood vessel dilation/constriction and blood pressure. **Other:** Any drug not falling into one of these subcategories.
Implanted/internal medical device	**Definition**: An implanted device that is worn inside the body, but does not provide electrical stimulation. This does not include software or assistive devices that are not worn, or worn on the outside of the body. The implanted device can either be permanent or removable. **Examples**: Indwelling catheters, bowel irrigation devices, recording or monitoring devices, colonic tubes, implanted array to monitor but not stimulate brain activity.
Nerve transfer/tendon transfer	**Definition**: A surgical procedure in which either nerves or tendons are surgically cut and transferred to another nerve or muscle.
Neuromodulation/electrical stimulation	**Definition**: An intervention in which electrical or magnetic stimulation is used to elicit activity of the nervous system. Electrodes or electrical fields can be used. The effect is that some part of the nervous system is stimulated. **Examples**: Functional electrical stimulation, epidural stimulation, peripheral nerve stimulation, transcranial magnetic stimulation, direct current stimulation, transcutaneous stimulation, transcranial stimulation with ultrasound.
Radiation therapy/laser therapy	**Definition**: Treatment with ionizing radiation, UV light, X-ray, or lasers.
Rehab/training/exercise	**Definition**: Any type of intervention comprised of exercise, activity-based training, or physical rehabilitation. **Examples**: Exoskeleton-mediated walking, treadmill training, stepping training, walking training, upper limb cycling, intermittent hypoxia, breathing training, high-intensity interval training, exercise regimens, passive motion exercises.
Surgical intervention/medical procedure	**Definition**: Surgical manipulations, surgical interventions, medical procedures, or procedure done during a spinal cord decompression surgery, except for nerve and tendon transfers. The surgery or procedure must be the primary intervention to be performed/evaluated. **Examples**: Surgical decompression, controlled surgical lesions of the nervous system, bladder surgeries, comparing or validating different methods of performing surgery, sustained induced hypertension/hypotension, hypothermia, bronchoscopy.
Other	**Definition**: Any intervention that does not clearly fit into the above categories. **Examples**: Passive heat stress, hypothermia, extracorporeal shockwave therapy, ischemic conditioning.

List of 14 classes of intervention used to classify spinal cord injury clinical trials. Each intervention type is defined and in some cases, examples of interventions are listed. Note that the “Drug” category encompasses 15 subcategories for different types of drugs and biological compounds.

**Table 2:** Outcome measure categories.

**Outcome type**	**Definition and examples**
Activity level	**Definition**: Assessments of physical activity level. **Examples**: Level of physical activity; the Physical Activity Scale for Individuals with Physical Disabilities (PASIPD); Physical Activity Questionnaire for People with Spinal Cord Injury (LTPAQ-SCI), International Physical Activity Questionnaire.
Autonomic dysreflexia	**Definition**: Adverse events resulting from overactivity of the autonomic nervous system in response to stimulation. This does not include autonomic function-related outcomes such as autonomic classification, autonomic control of respiratory or cardiovascular function.
Biomechanics/kinematics	**Definition**: Measurements of joint position, joint angles, torque, forces, and/or movement of the limbs during motor activity. **Examples**: Torque, resistance to stretching, degrees of flexion/extension of the arm or leg muscles, foot trajectory, propulsion, echogenicity ratio, load, contact time, muscle activity patterns during motion, joint forces.
Bladder function/bladder health	**Definition**: Measurements of bladder function or bladder health. **Examples**: Bladder filling, bladder voiding, bladder emptying, bladder pressure, compliance, leakage, frequency of urination, frequency of catheterization, neurogenic bladder, urinary tract infections.
Blood pressure/cardiovascular function	**Definition**: Measurements of blood flow, blood pressure, or heart function. **Examples**: Blood pressure, systolic blood pressure, hypotension, hypertension, heart rate, cerebral blood flow, arterial stiffness, Cerebral Vascular Resistance Index, VO_2_ peak (peak oxygen consumption), autonomic control of cardiovascular function, head-up tilt test, aerobic capacity.
Body mass/composition	**Definition**: Assessments of body mass or body composition. **Examples**: Body weight, body mass index, whole body skeletal muscle and fat mass, percentage of body fat, fat mass/fat-free mass.
Bone health	**Definition**: Assessments of bone health. **Examples**: Bone mineral density, bone health, bone mass, DXA scanning, osteoporosis, fracture, integral volumetric bone mineral content.
Bowel function/bowel health	**Definition**: Assessments of bowel function or health. **Examples**: Bowel function, bowel emptying, frequency of bowel movements, bowel management, bowel care routine, constipation, Knowles Eccersley Scott Symptom (KESS), Patient Assessment of Constipation Quality Of Life scale (PAC-QOL), Neurogenic Bowel Dysfunction (NBD) score.
Cognition	**Definition**: Assessments of cognitive ability. **Examples**: Memory, d2 Test of attention, any cognitive tests including, verbal learning test, word association tests, Stroop test, Cognitive Functioning as Measured by PASAT, Performance on Cognition Battery Tests, Performance on tests of information processing (WAIS-IV and Digit Span) and working memory (SDMT).
Depression/anxiety	**Definition**: Assessments of depression and/or anxiety. **Examples**: Depression symptoms, Anxiety symptoms, Hamilton Depression Rating Scale, HAM-D, 16-Item Quick Inventory of Depressive Symptomatology-Self Report (QIDS-SR16), Depression Scale of the Patient Health Questionnaire (PHQ-9), Change in Patient Health Questionnaire-9 for measure of patient depression severity.
Employment/occupational performance	**Definition**: Assessments or indices of employment or performance of occupational tasks. **Examples**: Ability to perform occupational tasks, rate or success in employment, perform work-related tasks, Canadian Occupational Performance Measurement (COPM).
Fatigue	**Definition**: Assessments of physical or cognitive fatigue or exertion level. **Examples**: Physical fatigue, cognitive fatigue, exertion level, perceived exertion, muscle fatigue.
Fertility/sexual function	**Definition**: Assessments of sexual function, sexual health, or fertility. **Examples**: Sexual health, sexual function, male sexual function, female sexual function, sexual quality of life, sexual dysfunction, fertility, sperm count, sperm viability, sperm health, ejaculation, erectile function, best method to obtain semen.
Independence	**Definition**: Assessments of the subject's level of independence in daily life. **Examples**: Independence, Spinal Cord Independence Measure (SCIM or SCIM-III), Spinal Cord Independence Measure-Self Reported (SCIM-SR), Craig Handicap Assessment and Reporting Technique (CHART), Functional Independence Measure (FIM), Wheelchair independence, performance of daily tasks.
Medical imaging	**Definition**: Non-invasive measurements of brain activity or anatomical parameters. **Examples**: Functional magnetic resonance imaging (fMRI), BOLD signal, MRI, X-ray, CT scan, DXA scan.
Metabolism	**Definition**: Assessments of body metabolism at the molecular level. **Examples**: Metabolic health, metabolism, resting metabolic rate, measurement of metabolites in the blood plasma or other body fluids, expression of gene products or metabolites, fasting insulin, fasting glucose, hemoglobin, insulin or glucose sensitivity, oxygen uptake, lipid measurements, circulating markers, inflammatory markers, blood assays, metabolic panels, energy expenditure.
Motor (lower extremities/locomotor function)	**Definition**: Assessments of lower body motor functions such as walking, ambulation, stepping, standing, or any other motor function of the lower extremities. **Examples**: Ten meter walk test, 6 minute walk test, WICSI-II, FIM gait score, Spinal Cord Injury Functional Ambulation Index (SCI-FAI), Berg Balance Scale (BBS), Lower-Extremity Motor Scores (LEMS), walking function, stepping function, standing, sit-to-stand.
Motor (not specified)	**Definition**: Assessments of motor function that are not specified as lower body, upper body, or trunk function. **Examples**: Strength, voluntary movement, task completion, physical function, motor function.
Motor (trunk)	**Definition**: Assessments of trunk motor function including trunk stability, trunk coordination, and sitting balance.
Motor (upper extremities/hand function)	**Definition**: Assessments of upper body and arm/hand motor functions. **Examples**: Graded Redefined Assessment of Strength, Sensibility, and Prehension (GRASSP) strength subscale, upper extremity muscle strength, Manual Muscle Testing (MMT), Hand Held Dynamometry (HHD), Grasp-Release Test, Activities of Daily Living Test, hand grasp, grip strength, upper motor strength, Disabilities of Arm, Shoulder, and Hand (DASH) scores, Michigan Hand Questionnaire (MHQ), Hand Function Tests.
Muscle and/or nerve function	**Definition**: Physiological assessments of muscle, nerves, and reflexes; not including motor functional outcomes. **Examples**: Muscle area, muscle cross-sectional area, motor evoked potentials (MEPs), H-reflex, nerve conduction velocity, muscle stretch reflexes, reflex activity, excitability, muscle activation, resting motor threshold (RMT), Physiology Measurements, electromyography (EMG), Single pulse transcranial magnetic stimulation, nerve action potential latency of nerve conduction studies.
Neurological score	**Definition**: This is a specific terminology that refers to the scores of a neurological exam or the level/degree of neurologic lesion. **Examples**: The ASIA impairment scale (AIS) score, the International Standards for Neurological Classification of Spinal Cord Injury (ISNC-SCI) exam.
Pain	**Definition**: Assessments of pain or pain relief. **Examples**: Pain reduction, Pain severity, Pain interference on quality of life, Mean Pain Intensity, Numeric Rating Scale (NRS), Neuropathic pain scale, International Basic Pain Dataset, mechanical allodynia, Patient-generated Index (PGI), Pain unpleasantness, Wheelchair User's Shoulder Pain Index (WUSPI), musculoskeletal pain.
Pharmacokinetics	**Definition**: Measurements of drug pharmacokinetics. **Examples**: Tolerability, blood serum and cerebrospinal fluid (CSF) levels of the drug, Pharmacokinetic (PK) profile, dosing concentration and drug levels over time, Area Under the Concentration-Time Curve.
Pressure injuries/pressure sores/wound healing	**Definition**: Measurements of pressure injuries, sores, or ulcers, or related parameters. **Examples**: Incidence of pressure ulcers/injuries/sores, wound healing, skin irritation, pressure on skin, bleeding.
Psychological/Social	**Definition**: Assessments of psychological and/or social health and well-being, not related to depression/anxiety. **Examples**: Mood, loneliness, Neuropsychological Tests, social integration, caregiver burden, social problem solving, self-esteem, life satisfaction, self-efficacy, social connectedness, perceived stress, The Ways of Coping Scale- Revised (WOC-R), Community Integration Questionnaire (CIQ), Stage of change Scales (SOC), resilience.
Pulmonary function/breathing/cough	**Definition**: Assessments of lung function, breathing, or cough. **Examples**: Pulmonary function, postoperative pulmonary complications, Lung volume, lung capacity, air flow, airway pressure, respiratory motor control, inspiratory/expiratory pressure, inspiratory/expiratory duration, inspiratory/expiratory function, autonomic control of respiratory function, forced vital capacity (FVC), peak inspiratory/expiratory flow, Exhaled Breath Condensate, forced expiratory volume, peak cough flow.
Quality of life	**Definition**: Questionnaires or surveys that allow the patient to self-assess their quality of life (QoL) and/or overall satisfaction with life. **Examples**: Quality of Life Index SCI version (QOLI-SCI), quality of life, satisfaction with life, Satisfaction with Life Scale (SWLS), Life satisfaction Checklist (LiSat-11), World Health Organization Quality of Life (WHQOL), RAND-36 questionnaire to measure health-related quality of life, Quality of Life on the SCI QL-23, EuroQoL.
Safety	**Definition**: This refers to the safety of the intervention being tested. Safety may be assessed by the number or frequency of adverse events (hospital visits, complications, infections, toxicity).
Sensory function	**Definition**: Assessments of sensory function or sensation anywhere in the body, except for pain. **Examples**: Pinprick sensory test (sharp vs. dull with a safety pin), touch sensory test (with a cotton ball), sensory discrimination, Sensation of urinary bladder filling, sensation in the legs, Thermal sensation, sensory examination, Graded Redefined Assessment of Strength, Sensation and Prehension (GRASSP), Semmes Weinstein monofilament sensation test.
Sleep	**Definition**: Assessments of sleep quality. **Examples**: Sleep quality, sleep apnea, apnea index.
Spasticity	**Definition**: Assessments of spasticity. **Examples**: Participant reported spasticity, severity of spasticity, Modified Ashworth Scale (MAS), Portable Spasticity Assessment Device (PSAD), Modified Penn Spasticity scale, Spinal Cord Injury Spasticity Evaluation Tool (SCI-SET).
Survival	**Definition**: Survival of patients at defined timepoints after treatment.
Thermoregulation	**Definition**: Measurements of body temperature and ability to regulate body temperature. **Examples**: Core Body Temperature, thermal comfort, skin temperature, sweating, thermal sensitivity.
Usability/feasibility/satisfaction of the intervention	**Definition**: Measurements of how well the intervention can be used by the patient. **Examples**: Device usability, level of assistance needed to use the intervention, success rate of task performance, Standardized Usability Questionnaire, any questionnaire that rates the ease of using the device, task completion time, System Usability Scale (SUS).
Wheelchair propulsion/mobility	**Definition**: Assessments of how well the patient is able to use a wheelchair. **Examples**: Wheelchair transfer, wheelchair mobility, Wheelchair Skills Test (WST), wheelchair propulsion test, wheelchair independence and mobility, 6-minute Push Test (6MPT), Wheelchair Outcome Measure (WhOM), figure 8 protocol (fatigue intervention).
Other	**Definition**: Any outcome that does not clearly fit into the above categories. **Examples**: Spinal alignment, spinal cord perfusion pressure, expression of genes or gene products, appraisal of disability, nutrition knowledge, skin moisture level.

List of 37 classes of outcome measure used to classify spinal cord injury clinical trials. Each outcome type is defined and examples of measurements or scores related to the outcome type are provided.

The 1,149 clinical trials that met our inclusion criteria were then annotated according to the types of interventions used and the types of primary and secondary outcomes assessed (Supplementary Table 1). For each trial, annotation was performed by at least two independent investigators. Only the information that was listed on the ClinicalTrials.gov webpage for a given clinical trial was used to categorize interventions and outcomes; no outside information (for example, information on other websites or published papers) was used to annotate trials. Interventions, primary outcome measures, and secondary outcome measures were annotated independently of each other, using the information available on the provided URL. If a clinical trial used multiple intervention types, each intervention type was listed once. For a given trial, if multiple outcome measures fell into the same category, that category was listed only once as an outcome for that trial. For example, a trial that lists several different measures of sexual function under Primary Outcomes on ClinicalTrials.gov would have “Fertility/sexual function” listed only once as a primary outcome type in our dataset. Primary and secondary outcomes are independent from one another, so it is possible that, e.g., “Fertility/sexual function” could be listed once under primary outcomes and once under secondary outcomes.

## Results

### General attributes and demographics of spinal cord injury clinical trials

**Figure 2:**
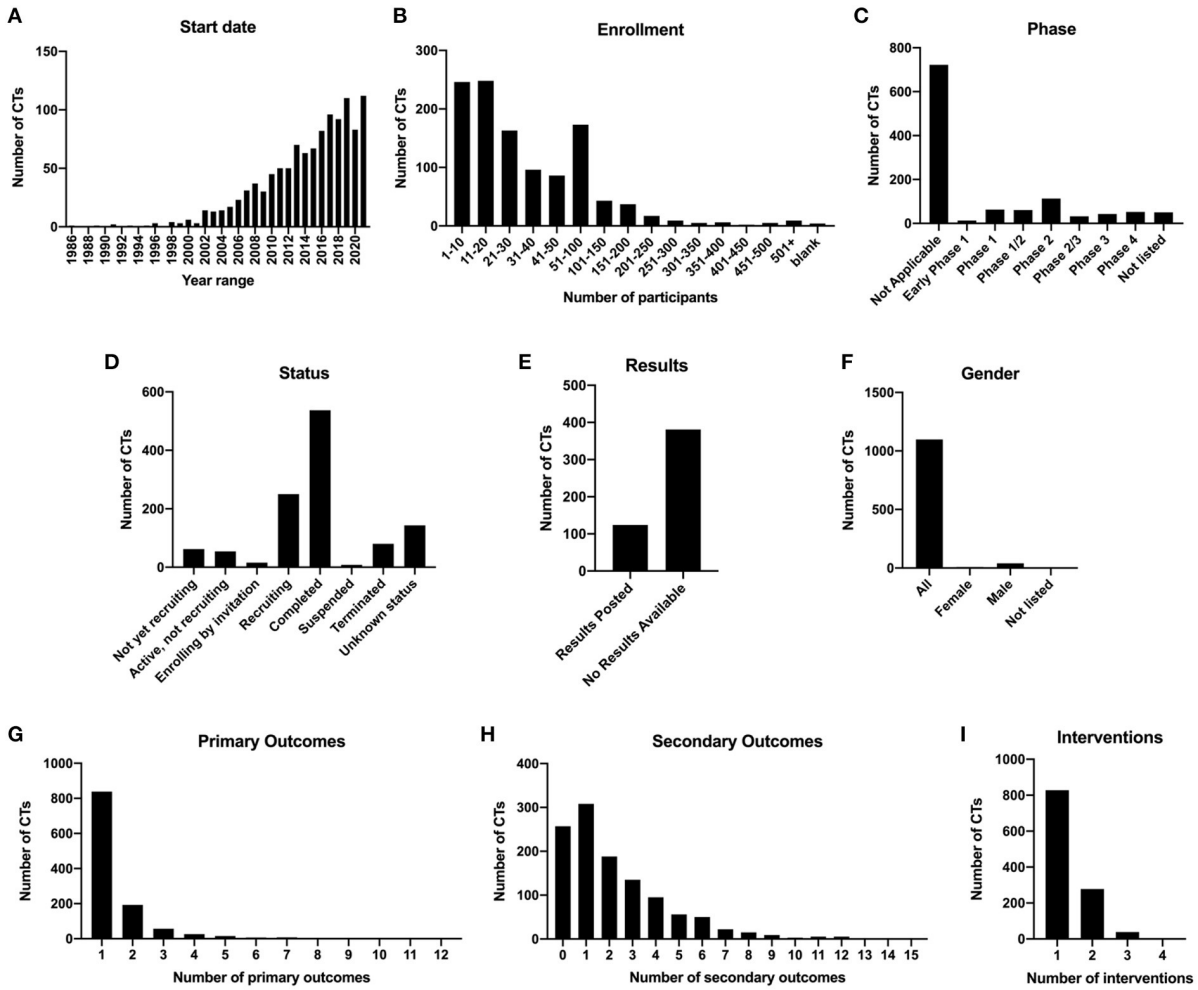
Demographics and statistics for 1,149 spinal cord injury clinical trials. **(A)** Numbers of clinical trials initiated per year from 1986 to 2021. **(B)** Number of clinical trials binned by actual or estimated enrollment of patients. **(C)** Number of clinical trials in each phase category. **(D)** Number of clinical trials in each status category. **(E)** Clinical trials marked as Completed and at least 1 year past the completion date, with results posted or no results available. **(F)** Number of clinical trials according to gender of enrolled subjects. **(G)** Number of clinical trials with 1, 2, 3, or 4 interventions. **(H)** Number of clinical trials with one or more types of primary outcome. **(I)** Number of clinical trials with one or more types of secondary outcome.

Of the 1,411 clinical trial listings identified, we excluded 262 trials that did not meet our eligibility criteria (Figure 1). We identified a total of 1,149 interventional clinical trials for spinal cord injury listed on ClinicalTrials.gov from 1996 to 2021, which we annotated according to types of intervention and outcome measures (Supplementary Table 1). We first analyzed general demographics and other attributes of the clinical trial data. We found that the numbers of new clinical trials per year have steadily increased over time, with 50% of all SCI clinical trials initiated between 2016 and 2021 (Figure 2A). In 2021, 112 new clinical trials were initiated, the most of any year in history.

We next analyzed enrollment. ClinicalTrials.gov lists either estimated enrollment or actual enrollment; however, it is not clear whether estimated enrollments were actually met for most listings, if results are not posted. The majority of clinical trials have low enrollments; 73.0% of trials had enrollment of 50 subjects or less (Figure 2B). Notably, only 9 of the 1,149 clinical trials had enrollment of over 500 participants. Among these were studies examining behavioral community wellness programs on the effects of lifestyle changes and transitions after injury (e.g., NCT03653390, “A Community Wellness Program for Adults Living With Long-term Physical Disability”; NCT02746978, “A Patient-centered Approach to Successful Community Transition After Catastrophic Injury”), as well as prospective studies examining the effects of surgical manipulations on outcomes such as survival rate (NCT01188447, “Evaluation of the Safety of C-Spine Clearance by Paramedics”; NCT03632005, “Negative Pressure Wound Therapy vs. Sterile Dressing for Patients Undergoing Thoracolumbar Spine Surgery”). Only three clinical trials ranked in the top 20 of enrollment are focused on testing the effects of experimental interventions (methylprednisolone, NCT00004759; minocycline, NCT01813240; methadone, NCT00006448) on neurological outcomes.

There are five phases of clinical trial, defined on ClinicalTrials.gov as “*Early Phase 1 (formerly listed as Phase 0), Phase 1, Phase 2, Phase 3, and Phase 4*.” Some trials were also listed as combined Phase 1/2 or combined Phase 2/3. According to the ClinicalTrials.gov website, “Not Applicable” describes “*trials without FDA-defined phases, including trials of devices or behavioral interventions*,” and this category should be chosen if the trial does not involve drugs or biological products (clinicaltrials.gov/ct2/about-studies/glossary). We found that 62.8% of trials were classified as “Not applicable,” and the second highest category was Phase 2, at 9.83% (Figure 2C). Fifty trials did not have any data listed for the Phase category (“Not listed”).

We further analyzed the types of intervention that were represented in each Phase of trial (Supplementary Figure 1). For trials that were classified as “Not applicable,” 42.4% involved rehab/training/exercise, 33.1% involved neuromodulation/electrical stimulation, 19.5% involved assistive/wearable technology, and 18.7% involved behavioral interventions. Surprisingly, 38 of these trials did involve drugs, cells, or biomaterials, so it is unclear how phase classification is not applicable to these trials. One strong trend is that the representation of the Drug category increases with advancing phase. For example, drug-related interventions represent 27.0% of Phase 1 trials, 64.6% of Phase 2 trials, 76.7% of Phase 3 trials, and 84.6% of Phase 4 trials (Supplementary Figure 1). Other interventions decrease with advancing phase; for example, cell or tissue transplantation represents 31.7% of Phase 1 trials, 14.2% of Phase 2 trials, but only 2.33% of Phase 3 trials and 0% of Phase 4 trials.

With regard to status, we found that 46.7% of the 1,149 trials were categorized as completed, whereas 23.1% were either recruiting or enrolling by invitation (Figure 2D). 10.1% of the 1,149 trials were not recruiting, and 7.66% were either suspended or terminated. Of the trials that were completed and at least 1 year post-completion date at the time of the search, 75.4% of them (381/505) had no results posted to ClinicalTrials.gov, whereas only 24.6% had results (Figure 2E). Of the 124 completed trials that had results, only 5 of those trials did not meet the primary endpoints; thus, 95.9% of completed trials with results posted were successful at meeting the primary endpoints. This information is indicated in Supplementary Table 1. When we analyzed gender, we found that the overwhelming majority (95.6%) of 1,149 clinical trials were targeted toward all genders, while 3.57% listed only males and only 0.78% listed only females (Figure 2F). Of the female-only trials, 8/9 of these were focused on women's health; for example, NCT02398331 “Sexual Health of Spinal Cord Injured Females” and NCT04872569 “Pilot Testing a Pregnancy Decision Making Tool for Women with Spinal Cord Injury”. Many of the male-only trials were focused on men's health, including reproductive and sexual health (10/41; NCT00223873, “The Use of Penile Vibratory Stimulation to Decrease Spasticity Following Spinal Cord Injury”; NCT00421983, “Efficacy and Safety of Tadalafil in Subjects with Erectile Dysfunction Caused by Spinal Cord Injury), catheterization (8/41; NCT02230540, “Intermittent Catheterization in Spinal Cord Injured Men”), or testosterone replacement therapy (7/41; NCT00266864, “Testosterone Replacement Therapy in Chronic Spinal Cord Injury”). A subset of male-only trials did not focus specifically on men's health (NCT02703883, “Body Weight Support in Spinal Cord Injury”; NCT01274975, “Autologous Adipose Derived MSCs Transplantation in Patient With Spinal Cord Injury”).

### Representation of intervention and outcome types

Types of primary and secondary outcomes were also analyzed. Outcome types are listed in Table 2. We found that the majority of the 1,149 trials (73.0%) examined 1 type of primary outcome, 16.8% examined 2 types of primary outcomes, and 4.96% examined 3; the remaining 5.22% of trials examined 4 or more types of outcomes, with a maximum of 12 types of primary outcomes tested in a single trial (Figure 2G). Inclusion of a single primary outcome in most of these studies is consistent with the goal of addressing a focused research question (Vetter and Mascha, 2017), while inclusion of multiple primary outcomes can inflate the false positive rate (Othus et al., 2022). For secondary outcomes, most trials (26.8%) examined only 1 type, though 22.4% did not examine any secondary outcomes (Figure 2H). 34.5% of trials examined 3 or more types of secondary outcomes, with a maximum of 15 types in a single trial.

We next analyzed the numbers of intervention types and outcome types per trial. Intervention types are listed in Table 1. Of the 1,149 clinical trials, 72.1% listed only one intervention, and 24.2% listed two interventions; <5% of trials listed 3 or 4 interventions (Figure 2I). Of the clinical trials testing more than one intervention, 74.8% of these featured Rehab/training/exercise as one of the interventions. Top combinatorial interventions included Assistive/wearable technology + Rehab/training/exercise (25.5%), and Neuromodulation/electrical stimulation + Rehab/training/exercise (34.6%). Four trials had 4 interventions; for example, NCT02136823, “Impact of Persistent Conductances on Motor Unit Firing in SCI,” tested the effects of three different drugs plus a stretching exercise on muscle reflex excitability.

**Figure 3:**
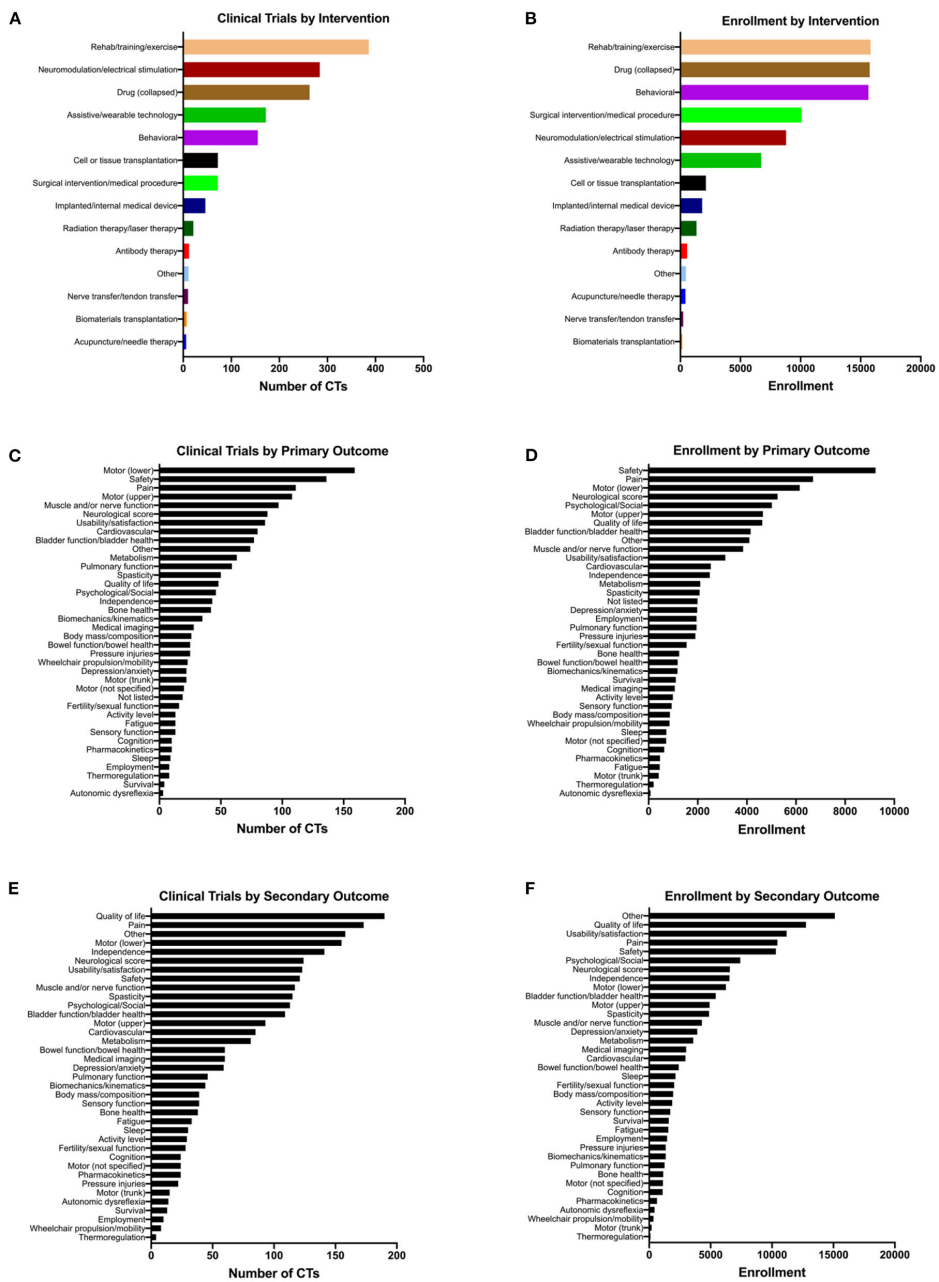
Therapeutic spinal cord injury clinical trials classified according to intervention and outcome types. Note that a given trial may have more than one intervention and multiple outcomes, so the total numbers of clinical trials in **(A,C,E)** add up to more than 1,149. **(A)** The total number of clinical trials for each class of intervention. **(B)** The cumulative enrollment for all clinical trials that use each type of intervention. **(C,E)** The total number of clinical trials listing each type of **(C)** primary and **(E)** secondary outcome. **(D,F)** The cumulative enrollment for all clinical trials that list each type of **(D)** primary and **(F)** secondary outcome.

We sought to quantify the number of clinical trials according to the types of intervention used, and the types of outcomes assessed. We first quantified the number of the 1,149 trials that used each of 28 classes of intervention, with Drug subcategories collapsed (Figure 3A). We found that the highest-ranking category was Rehab/training/exercise with 386 clinical trials, followed by Neuromodulation/electrical stimulation (284 trials), Drug (all categories; 263 trials), Assistive/wearable technology (172 trials), and Behavioral (155 trials). We further broke down the Drug category into 15 sub-categories and found that neuromodulatory drugs were the most highly represented (70 trials) (Supplementary Figure 2). In addition to ranking interventions by the number of trials, we also calculated total human subject enrollment in all of the trials utilizing each intervention type (Figure 3B). Using this approach, Rehab/training/exercise and Behavioral ranked highest with 15,824 and 15,650 enrolled, respectively. Drug (all subcategories; 15,753 enrolled) also had among the highest enrollments of any intervention. Some of the lowest categories by enrollment are Biomaterials transplantation (150), Nerve transfer/tendon transfer (237), and Acupuncture/needle therapy (421).

The primary outcomes associated with the greatest number of the 1,149 clinical trials were Motor (lower extremities/locomotor function) with 159 trials, Safety with 136 trials, Pain with 111 trials, and Motor (upper extremities/hand function) with 108 trials (Figure 3C). Among the least-represented primary outcomes were Autonomic dysreflexia (3 trials), Thermoregulation (8 trials), and Sleep (9 trials). Upon calculating total enrollment for primary outcomes, we found that the highest enrollments were associated with Safety with 9,236 enrolled, Pain with 6,692 enrolled, Motor (lower extremities/locomotor function) with 6,147 enrolled, and Neurological score with 5,249 enrolled (Figure 3D). Autonomic dysreflexia was still the lowest-ranked outcome by enrollment, with only 77 subjects enrolled in trials that evaluated it as a primary outcome measure. For secondary outcomes, we found that Quality of life was listed for the greatest number of trials (190 trials), followed by Pain with 190 trials, Other with 158 trials, and Motor (lower extremities/locomotor function) with 155 trials (Figure 3E). Upon analyzing actual enrollment associated with secondary outcome measures, we found that there was much greater enrollment represented for secondary outcomes; the highest-ranked categories were Other with 15,115 enrolled, Quality of life with 12,765 enrolled, Usability/feasibility/satisfaction with 11,188 enrolled, and Pain with 10,438 enrolled (Figure 3F). This reflects the finding that trials were likely to have a greater number of secondary outcomes listed compared to primary outcomes (Figures 2G,H).

### Trends in interventions and outcomes over time

**Figure 4:**
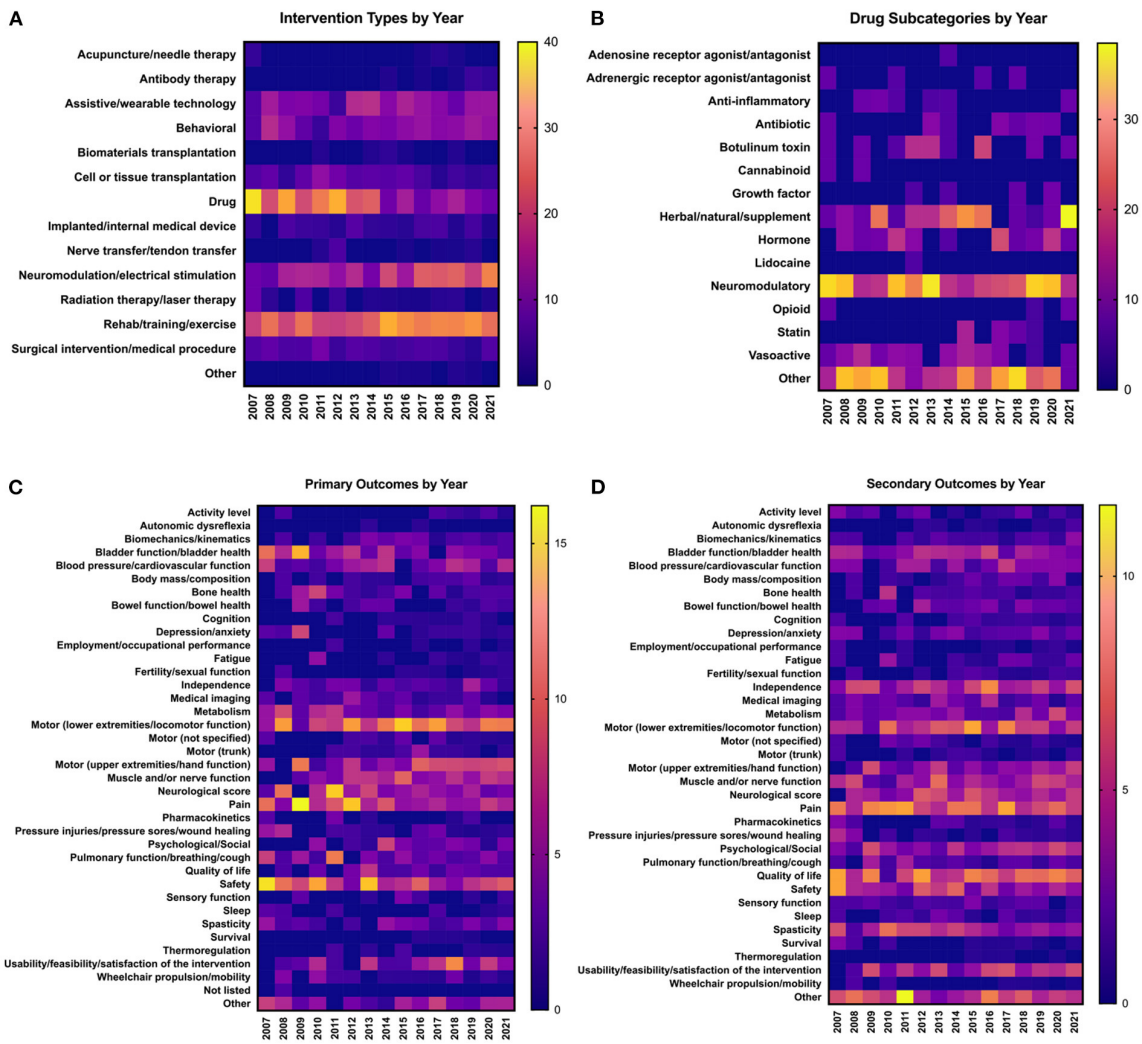
Trends in clinical trial interventions and outcomes over time. Data are from clinical trials initiated between 2007 and 2021. All data are represented as percentages of the trials in a given year that utilize each type of **(A,B)** intervention or **(C,D)** outcome; values in individual columns add up to 100%. **(A)** Frequency of types of interventions used in clinical trials each year. **(B)** Breakdown of the types of drugs that make up the “Drug” category in **(A)**. Values in individual columns add up to 100% of total drugs in a given year. **(C)** Frequency of types of primary outcome measures assessed each year. **(D)** Frequency of types of secondary outcome measures assessed each year.

We next sought to understand how interventions and outcomes have changed over time. Because of limited data availability for clinical trials initiated prior to 2007, we elected to focus on analyzing trends in data over the past 15 years, from 2007 to 2021. We first analyzed trends in interventions tested over time. In 2007, drugs/biological compounds were the most represented intervention, with 37.8% of total interventions falling into this category (Figure 4A). However, over time there has been a gradual decrease in the proportion of interventions that are drugs; most recently in 2021, only 8.02% of all interventions were drugs. Figure 4B shows the breakdown of different subcategories of drugs comprising the “Drug” category. In most years, neuromodulatory, herbal/natural, and “Other” subcategories represent the greatest contribution to the Drug category. While most types of interventions have remained relatively stable over time, the Neuromodulation/electrical stimulation and Rehab/training/exercise categories have increased over time (Figure 4A). In 2021, Neuromodulation/electrical stimulation represented 27.8% of all interventions, and Rehab/training/exercise represented 25.3% of all interventions. In 2021 alone, 112 new clinical trials were initiated (Figure 2A); of these, 45 utilize Neuromodulation/electrical stimulation, and 41 utilize Rehab/training/exercise. In the past 5 years (2017–2021), 162 new clinical trials for Neuromodulation/electrical stimulation and 190 new trials for Rehab/training/exercise were initiated.

We did not detect many major shifts in the representation of primary and secondary outcome measures over time (Figures 4C,D). Some general trends emerged; for example, primary outcomes such as lower extremity motor function have stayed relatively steady over time, whereas upper extremity motor function has gradually increased (Figure 4C). Some primary outcome measures, such as autonomic dysreflexia, thermoregulation, and depression/anxiety, have remained consistently underrepresented compared to other outcome measures. For secondary outcome measures, some have remained consistently high over the past 15 years, such as pain, independence, and quality of life (Figure 4D). Overall, the representation of most secondary outcomes has remained relatively stable. Together, these data reveal that representation of primary and secondary outcomes has remained relatively stable over time.

## Discussion

### Emerging trends in SCI clinical trials

Of all the 1,149 clinical trials we reviewed, we observed that the majority of these enrolled <100 participants (Figure 2B). The number of participants enrolled in a clinical trial is uniquely based on the design of the trial, phase of the trial and therapeutic being tested. Note that higher recruitment will be needed to sufficiently power the study (Bracken et al., 1997; Fawcett et al., 2007). Enrollment of clinical trials specifically for SCI present challenges such as low incidence of injury, variable injury/severity among each participant, highly debatable approaches regarding therapeutic intervention and high cost of enrolled participants (Mulcahey et al., 2020). Several studies have examined these challenges of recruitment and the difficulties of maintaining recruitment in clinical trials and has opened the discussion for adaptive trial designs (Chow and Chang, 2008; Dragalin, 2011; Meurer et al., 2012; Meurer and Barsan, 2014; Bauer et al., 2016; Blight et al., 2019; Hubli et al., 2019; Kwon et al., 2019; Seif et al., 2019; Mulcahey et al., 2020).

Notably, we found that 72% of SCI clinical trials employed only one intervention (Figure 2I). It is a common consensus that to combat the complex nature of SCI, there will be no “magic bullet” single treatment; rather, effective therapies will likely be combinatorial in nature (Bunge, 2001; Ramer et al., 2005; Hawryluk et al., 2008; Olson, 2013; Griffin and Bradke, 2020). Of the 28% of trials using more than one intervention, almost 75% of these employed rehab/training/exercise as one of the interventions. Furthermore, only 5.1% of these combinatorial trials are either Phase 3 or Phase 4 studies. Hence, this data indicates a need to progress toward advancement of combinatorial clinical trials to combine the most promising therapies. Scientists and clinicians now face the challenge of figuring out how to incorporate rigor into study design while testing the greatest number of therapeutics in combination.

According to ClinicalTrials.gov, “*Primary and secondary outcomes are required by law to be analyzed and reported if any data was collected for the outcome. The primary and secondary endpoints should be pre-specified*”. The primary outcome is the outcome measure of greatest importance and usually the one used in the power calculation during clinical trial design. The highest-ranked categories in primary outcome are motor (lower extremities/locomotion), safety, and pain while the lowest ranked are autonomic dysreflexia, thermoregulation, and sleep (Figure 3C). Similarly, the highest ranked categories of primary outcome also have the highest enrolled participant totals, while autonomic dysreflexia also has the lowest number of enrolled participants (Figure 3D). A natural question, therefore, is, “Does this reflect the priorities of the SCI community” (Anderson, 2004)? However, this is a difficult question to answer. It is clear that the expressed needs and priorities change from person to person, and are dependent on a variety of factors such as injury level, severity, and time after injury (i.e., acute or chronic) (Glass et al., 1991; Anderson, 2004; Simpson et al., 2012; Trezzini and Phillips, 2014; Zanini et al., 2021).

### Trends over time

Over the past 15 years, clinical trials have undergone some notable shifts in the representation of intervention and outcome types. It is important to note that clinical trial records may be incomplete prior to September 2007, when registration and submission of clinical trials and study results with ClinicalTrials.gov first became legally mandated through Section 801 of the Food and Drug Administration Amendments Act (FDAAA 801; clinicaltrials.gov/ct2/manage-recs/fdaaa), with the exception of phase 1 drug investigations, small clinical trials to determine feasibility, and certain clinical trials to test prototype devices (prsinfo.clinicaltrials.gov/ACT_Checklist.pdf). Hence, this could result in artificially low numbers prior to 2008, as there were likely more trials being conducted than were registered to ClinicalTrials.gov. Another consideration is that beginning in 2004, the International Committee of Medical Journal Editors (ICMJE) have required any interventional human trials to be registered at ClinicalTrials.gov as a prerequisite for publication (clinicaltrials.gov/ct2/manage-recs/background).

Beginning in 2007, the most represented intervention category was “Drug,” mainly comprised of neuromodulatory drugs; this may explain why most clinical trials in advanced phases are drug-related. As the representation of drug-based interventions has gradually decreased over time, there were concomitant increases in both rehab/training/exercise and neuromodulation/electrical stimulation (Figure 4A). This increase undoubtedly reflects advancements in technology allowing novel engineering of neuromodulation/electrical stimulation and a widely accepted consensus that rehabilitation is fundamental to improved outcomes (Whalley Hammell, 2007; Gomara-Toldra et al., 2014). An example of this is the combination of assistive technology (e.g., exoskeletons) with rehab/training/exercise. In 2014, the FDA approved the first robotic exoskeleton, ReWalk (ReWalk Robotics, Inc.) (Zeilig et al., 2012; Miller et al., 2016; Ahuja et al., 2017b). As noted above, hundreds of new clinical trials testing neuromodulation- and rehabilitation-based interventions have been initiated in the past few years alone. If this trend continues, the future of clinical SCI research will be overrepresented with these types of interventions.

Although some outcomes—for example, bladder function/health as a primary outcome—appear to be have decreased representation over time (Figure 4C), this is not due to a net reduction in bladder trials. For example, from 2007 to 2021 there has been an average of 4.2 ± 2.1 clinical trials measuring bladder function/health as a primary outcome per year, with 4 trials in 2007 and 4 trials in 2021 (Supplementary Table 1). In other words, the total numbers of trials measuring bladder function/health are not decreasing over time, but as the number of total clinical trials grow, bladder outcomes are not keeping up. This is also true for trials measuring pain as a primary outcome; representation of pain appears to decrease over time, but studies have actually increased from 4 trials in 2007 to 11 trials in 2021 (Supplementary Table 1). It is important to consider these trends in light of the challenges faced by the SCI community; for example, pain was ranked as the #1 most frequently cited challenged faced by those living with SCI according to a recent NASCIC survey (North American Spinal Cord Injury Consortium, 2019).

### Gaps in clinical trial reporting

ClinicalTrials.gov was developed in an effort to make all ongoing trials accessible to clinicians and patients, combat publication bias, and enhance transparent reporting of clinical trials (Dickersin and Rennie, 2003). This website is a valuable data source, allowing users to track and evaluate the progression of clinical trials in a centralized repository with mandated regulations for reporting results (Zarin et al., 2011). This database also allows ease of systematic analyses elucidating trends in clinical trial design and in therapeutic interventions, as others have done previously in different fields (Hirsch et al., 2013; Jaffe et al., 2019; Wortzel et al., 2020). Our analyses clearly demonstrate that there are gaps in reporting including a lack of clarity with regard to categorizing trials as “interventional,” reporting the specific characteristics of the SCI itself, or reporting of study results. More broadly, multiple studies have identified areas for potential improvement in reporting and usability for ClinicalTrials.gov (Wu et al., 2016; Chaturvedi et al., 2019; Warner et al., 2021). In 2021, Warner et al. conducted a systematic analysis on a subset of data extracted from spinal cord injury clinical trials; the authors identified key areas of improvement in reporting of these clinical trials (Warner et al., 2021). For instance, only 11.2% of trials correctly identified their study type, provided valid study status and provided sufficient detail about injury characteristics (Warner et al., 2021).

In our analysis, gaps in reporting became apparent during systematic review of clinical trial characteristics. One of the most noteworthy examples is that although almost half of clinical trials were marked as “Completed,” 75.4% of completed trials have no results available on ClinicalTrials.gov (Figures 2D,E). This is similar to a previous finding that only 23.5% of 344 SCI trials with “Completed” status had results posted on ClinicalTrials.gov (Warner et al., 2021). However, we found that the absence of posted results did not necessarily mean that results from the study were not available elsewhere. We performed a PubMed search of 50 randomly selected trials that are listed as “Completed” with “No results available,” and found that 27 of 50 (54%) of these trials had published results associated with the study outcomes. ClinicalTrials.gov denotes that “when results are not available for a study, the results tab is labeled “No Results Posted.” Results of a study may not be posted for the following reasons: the study may not be subject to U.S Federal requirement to submit results, the deadline for results submission has not passed or the submission of results information has been delayed by the submission of a certification or a request to extend the results submission deadline” as per the FDAAA 801 Final Rule (clinicaltrials.gov/ct2/about-site/history). This issue of reporting is not new and has been observed by authors of other meta-analyses based on ClinicalTrials.gov data (Anderson et al., 2015; Warner et al., 2021). It is crucial that the public, scientific and clinical community be able to see results of clinical trials so that informed decisions can be made moving forward and integrated into the decision of participation, funding and approval of future clinical trials. Working with incomplete datasets leaves individuals unequipped to judge the novelty or innovation of future trials and can directly contribute to redundancy of clinical trials. To remedy this, we join others in suggesting that reporting publications and trial results to ClinicalTrials.gov should be required as part of clinical trial reporting standards (publications.parliament.uk/pa/cm201719/cmselect/cmsctech/1480/148002.htm).

These gaps in reporting underscore a need for better reporting standards and more transparent data sharing. Several studies have demanded that clinical trial results be open access (Kramer et al., 2017) and have recommended that efforts be made to harmonize/standardize data elements so that comparisons between trials can be made (Landis et al., 2012; Steward et al., 2012; Lammertse, 2013; Lemmon et al., 2014; Ahuja et al., 2017b; Gensel and Orr, 2021). Several initiatives have been established to enhance data sharing such as the creation of Open Data Commons-SCI (ODC-SCI) enabling FAIR Sharing practices (Biering-Sorensen et al., 2015; Callahan et al., 2017; Mulcahey et al., 2017; Fouad et al., 2020a), the development of TRACK-SCI (Transforming Research and Clinical Knowledge in SCI) (Tsolinas et al., 2020), the North American Clinical Trials Network SCI Registry (Grossman et al., 2012), the International Spinal Cord Society SCI Data Sets (DeVivo et al., 2006) and the National Spinal Cord Injury Statistical Center Database (DeVivo et al., 2002).

### Perspectives from the clinician-scientist

In most cases, the burden of reporting falls on the clinician-scientists at the institution conducting the clinical trial (Tse et al., 2009). Some institutions have supported the creation of administrative positions dedicated to clinical trials reporting to ease the burden of the primary investigator. However, in our experience, the greater challenge lies in the strict formatting of outcomes required by ClinicalTrials.gov. Whereas, an Institutional Review Board can manage a variety of formatting, allowing for investigators to use language directly from a grant application, this is not available in ClinicalTrials.gov. This may directly impact data analysis because results for the funding agency is the priority. Similarly, results for a manuscript may take precedence over the results requested by ClinicalTrials.gov. Another obstacle is that clinicians are often asked to fill out required information in such a way that meets the website's standard but does not necessarily require important information (for example, we observed that several registered clinical trials left fields as “not listed,” “unknown status” or “blank,” see Figure 2 and Supplementary Table 1). This lack of “policing” has contributed to this incomplete data set where several trials do not have results posted or have left important information as inaccurately listed. It has become apparent that there needs to be a call for standardizing and updating these reporting standards. It could be beneficial to link IRB permitting with the ClinicalTrials.gov website thereby allowing more accurate reporting of data while also easing the paperwork burden on clinicians. Additionally, having IRB mandate reporting of results with permit renewal to ClinicalTrials.gov could present an avenue to enhance reporting of results.

### Perspectives from the SCI community

SCI research and clinical trials have been conducted for several decades, yet there remains no FDA approved, proven effective treatment for any outcomes associated with SCI; available treatment options are limited, and there is continuing debate about the standard level of care. There has been justifiable frustration and apathy expressed by individuals living with SCI in reaction to the promise of treatments being “just around the corner” fueled by media hype, as well as the slow pace of translation after decades of pre-clinical research (Kwon et al., 2010).

Individuals with SCI have made clear their desire to be involved in the research process from start to finish (Morse et al., 2021). In a 2019 study by the North American Spinal Cord Injury Consortium, community members ranked their highest priorities as receiving research information and serving as advisors to research teams (North American Spinal Cord Injury Consortium, 2019). This brings up two important topics of discussion: inclusion of lived experience consultants and accessibility of research to this population. As a direct result of this continuing call for inclusivity in research, some funding agencies such as the Department of Defense SCI Research Program and the Paralyzed Veterans of America Research Foundation have included individuals living with SCI as peer reviewers on their grant review panels and have required new grant submissions to include SCI consumer advocates or lived experience consultants to partner with research laboratories (Anderson, 2021). Additionally, several institutions strongly encourage the development of partnership between researchers and SCI community.

With regard to accessibility of research, many barriers remain present. One major example that this review brings to attention is that although 76.5% of SCI clinical trials do not have results posted to ClinicalTrials.gov, it is often the case that if and when published results are posted, they are still inaccessible to general public due to subscription requirements for journal access. This is a major issue because if results are posted on ClinicalTrials.gov they are primarily in tabular format and lack interpretation that is present in peer-reviewed publications. *It is critically important for SCI community members to be able to access and interpret clinical trial data*. They need to be able to understand what types of clinical trials are ongoing, be able to determine whether there are any they are eligible for, and access/look at results so they can interpret results for themselves. Resources such as scitrials.org and scitrialsfinder.net are working toward this goal. It would be useful, for example, if the national clinical trial registry developed a systematic process for suggesting clinical trials tailored to individuals based on profile suitability rather than consumer demand. To date, “*ClinicalTrials.gov* *is designed to benefit the general public by expanding access to trial information*” (Zarin et al., 2011), yet we found that this dataset was incomplete and will likely be inaccessible to the general public.

Finally, we have identified some actionable items that, if implemented, could be useful for improving the usefulness of clinical trial data to the SCI community. First, a designation labeling interventional SCI trials as “therapeutic” vs. “not therapeutic” would be helpful; we found that 2.62% of SCI clinical trials labeled as “Interventional” were not actually testing a therapeutic intervention (Figure 1), and it would be useful for SCI community members to easily identify trials of therapeutics. Second, some clarification would be useful regarding future planned trials associated with a given intervention, and expectations for future clinical translation. We found that inconsistent or inaccurate application of FDA phase status, as well as the absence of sequential or graduated trial strategies, suggest that most trials do not appear to be designed to progress toward FDA approval. Additionally, it is unclear how much conceptual or programmatic overlap exists among clinical trials testing very similar interventions (e.g., neuromodulatory interventions for locomotor recovery), so some cross-referencing to indicate relationships between trials that are testing the same device, or trials that are otherwise linked in scope, would be useful. Finally, as a future goal, some integration of ClinicalTrials.gov with major data sharing initiatives would be a useful approach to recognize synergies between studies and improve clinical trial design moving forward into the future.

## Conclusion

This systematic review provides a comprehensive view of SCI interventional clinical trials. The number of new SCI clinical trials initiated each year continues to climb. A large proportion of new trials are focusing on interventions such as neuromodulation, electrical stimulation, and rehabilitation. Over time, trials testing drug-based interventions have decreased in representation. These findings should be useful to scientists, clinical researchers, and the SCI community as a resource for understanding the trends in, and evolution of, interventional SCI clinical trials. However, gaps in reporting to ClinicalTrials.gov may present barriers that will limit the usefulness of this data to the public, scientific, and clinical communities. There is a need for improving reporting standards to ClinicalTrials.gov.

## Data availability statement

The original contributions presented in the study are included in the article/Supplementary material, further inquiries can be directed to the corresponding author/s.

## Author contributions

VD and JD conceived of the study, performed study classification and data analysis, and wrote the manuscript. NR, KK, SM, and MP performed study classification. CB performed data analysis and contributed to manuscript writing. JC, SL, KN, PN, MR, CG, and AS contributed to study design and manuscript writing. All authors contributed to the article and approved the submitted version.

## Conflict of interest

The authors declare that the research was conducted in the absence of any commercial or financial relationships that could be construed as a potential conflict of interest.

## Publisher's note

All claims expressed in this article are solely those of the authors and do not necessarily represent those of their affiliated organizations, or those of the publisher, the editors and the reviewers. Any product that may be evaluated in this article, or claim that may be made by its manufacturer, is not guaranteed or endorsed by the publisher.
