# Supplementary material for: Fighting for recovery on multiple fronts: The past, present, and future of clinical trials for spinal cord injury
Source: Front Cell Neurosci. 2022 Sep 7;16:977679. doi: 10.3389/fncel.2022.977679 (PMC9533868; doi:10.3389/fncel.2022.977679)
Supplement: Supplementary file 1 [file Data_Sheet_1.docx]

**Legend for Supplementary Table 1 (spreadsheet).**

**Sheet 1** is a table of 1,411 spinal cord injury clinical trials including all raw data downloaded from ClinicalTrials.gov. **Sheet 2** is a table of 262 trials that were excluded from this study, with the reason for exclusion in column AA. **Sheet 3** is a table of the remaining 1,149 SCI clinical trials testing a therapeutic intervention that were included for analysis. For Sheet 3, columns A through Z are raw data downloaded directly from ClinicalTrials.gov. Columns AA through BF are annotations added by the authors.


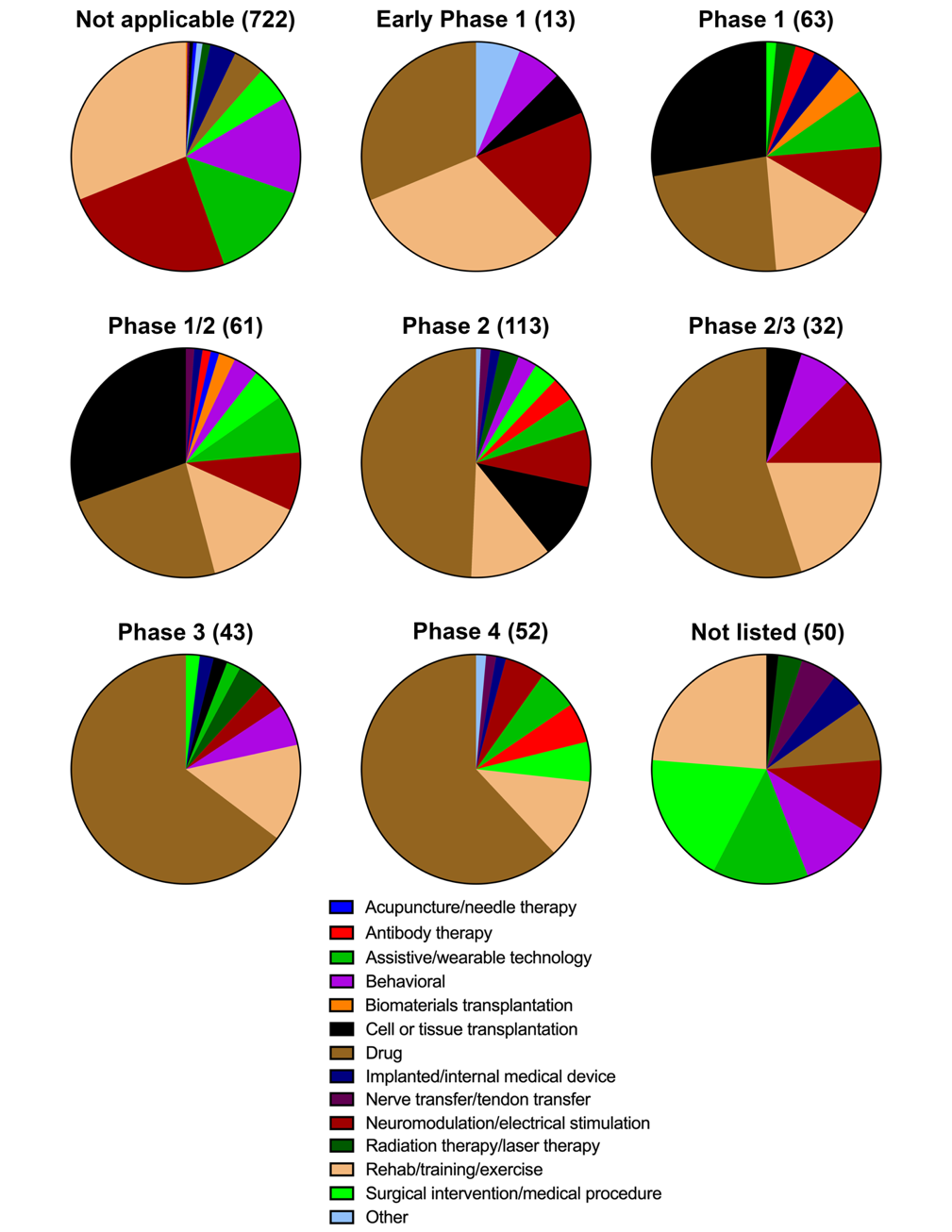


**Supplementary Figure 1. Breakdown of trials by phase category.**

The total number of trials in each phase are included in parentheses above each pie chart. The pie charts represent the fraction of trials in each phase that utilize the corresponding interventions.


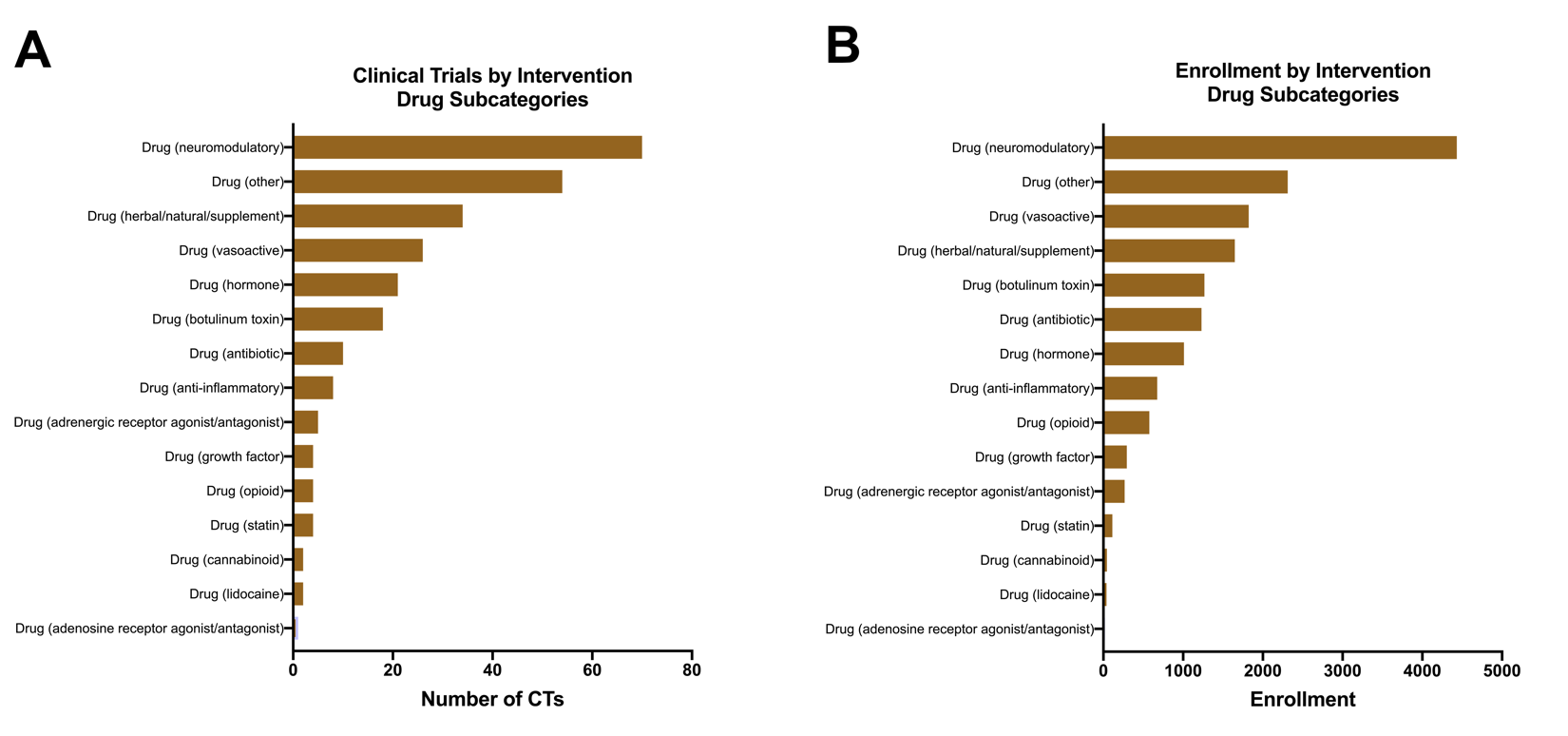


**Supplementary Figure 2.** These graphs show the expanded data for the Drug subcategories in Figure 3A-B. (**A**) The total number of clinical trials for each class of drug-related intervention. (**B**) The cumulative enrollment for all clinical trials that use each type of drug-related intervention.
